# Factors Associated With Self-reported Symptoms of Depression Among Adults With and Without a Previous COVID-19 Diagnosis

**DOI:** 10.1001/jamanetworkopen.2021.16612

**Published:** 2021-06-11

**Authors:** Roy H. Perlis, Mauricio Santillana, Katherine Ognyanova, Jon Green, James Druckman, David Lazer, Matthew A. Baum

**Affiliations:** 1 Departmentof Psychiatry, Massachusetts General Hospital, Boston; 2 Departmentof Psychiatry, Harvard Medical School, Boston, Massachusetts; 3 Computational Health Informatics Program, Boston Children’s Hospital, Boston, Massachusetts; 4 Department of Pediatrics, Harvard Medical School, Boston, Massachusetts; 5 Department of Epidemiology, Harvard T.H. Chan School of Public Health, Boston, Massachusetts; 6 Departmentof Communications, Rutgers University, New Brunswick, New Jersey; 7 Network Science Institute, Northeastern University, Boston, Massachusetts; 8 Departmentof Political Science and Institute for Policy Research, Northwestern University, Evanston, Illinois; 9 Kennedy School of Government, Harvard University, Cambridge, Massachusetts

## Abstract

This survey study compares features of self-reported symptoms of major depression in adults with or without a prior COVID-19 diagnosis.

## Introduction

Rates of major depressive symptoms are elevated after acute infection with SARS-CoV-2.^1,2,3^ A key question is whether such symptoms represent a general consequence of stress associated with acute illness or whether they reflect more specific sequelae associated with COVID-19 pathophysiology itself. To examine this possibility, in this survey study, we compared features of major depression in individuals with or without prior COVID-19 illness.

## Methods

We conducted 12 waves of an internet nonprobability Qualtrics survey using a multipanel commercial vendor (PureSpectrum) approximately every month between May 2020 and February 2021 for individuals aged 18 years and older. The study was approved by the institutional review board of Harvard University. All participants provided written informed consent. We followed the American Association for Public Opinion Research (AAPOR) reporting guideline.

The survey included sociodemographic questions on sex, income, age, education, urbanicity, and self-identified race/ethnicity using 5 US Census categories. Race and ethnicity were included because a goal of the study is to understand how these and other sociodemographic features affect responses to COVID-19 illness and quarantine. Respondents indicated whether and when they had received a clinician diagnosis of COVID-19 illness or a positive SARS-CoV-2 test result. Participants also completed the Patient Health Questionnaire (PHQ)–9 as a measure of depressive symptoms,^4^ with a score of 10 or higher defined as at least moderate symptoms.

We applied multiple logistic regression with moderate or greater depressive symptoms as the dependent variable and sociodemographic features as independent variables, testing an interaction term with prior COVID-19 for each feature, using *glm* in R statistical software version 3.6 (R Project for Statistical Computing). We compared mean values for each depressive and anxious symptom between individuals with or without prior COVID-19 using 2-sided *t* tests. Significance was set at *P* < .05. Finally, we examined prevalence of depressive symptoms by months elapsed since COVID-19.

## Results

Among 91 791 individuals who completed the PHQ-9, 61 472 (67.0%) were female, 9667 (10.5%) were Black, 6686 (7.3%) were Hispanic, 5306 (5.8%) were Asian, 36 818 (40.1%) completed some college, 22 216 (24.2%) were in urban locations, and 16 158 (17.6%) were rural. Their mean (SD) age was 42.34 (16.36) years, their median (interquartile range) annual income was $49 000 ($22 500-$85 000), 5945 (6.5%) reported prior COVID-19 clinician diagnosis or test result, and 28 617 (31.2%) reported moderate or greater depressive symptoms.

**Figure 1.**
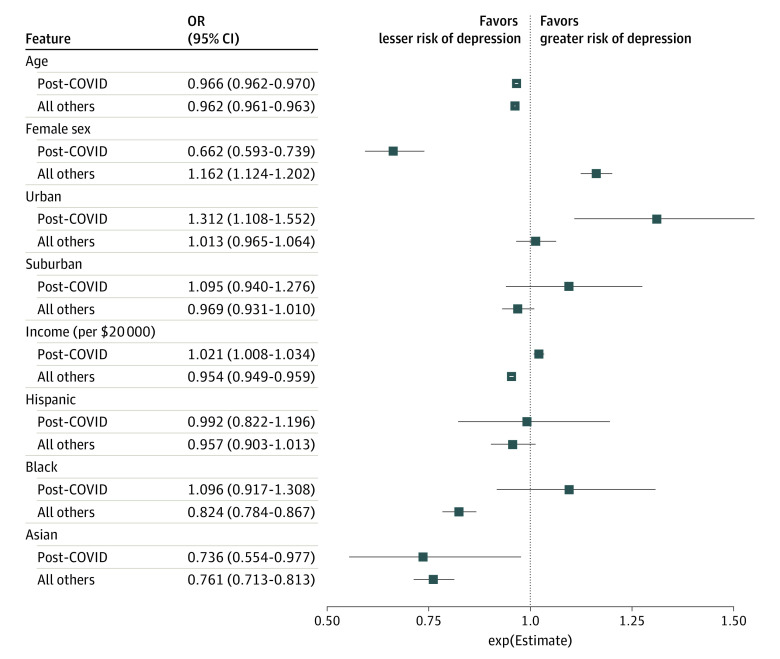
Logistic Regression Model for Association of Sociodemographic Features With Moderate or Greater Depressive Symptoms, Among Adults With or Without Prior COVID-19 Illness

**Figure 2.**
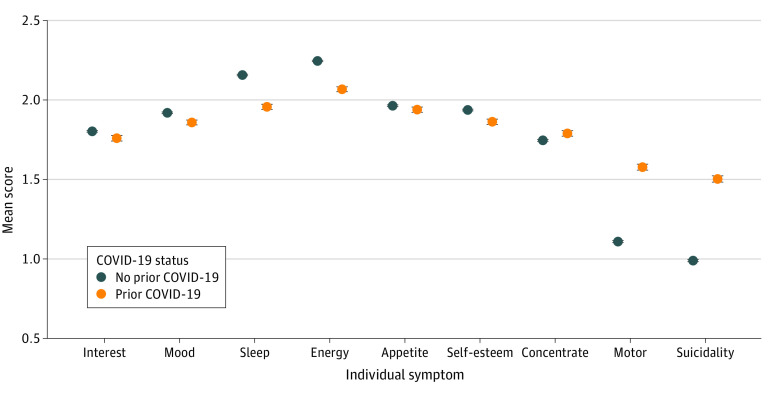
Individual Patient Health Questionnaire–9 and Patient Health Questionnaire–4 Symptoms Among Adults With Current Moderate or Greater Depressive Symptoms, With and Without Prior COVID-19 Infection

In regression models for depressive symptoms, significant associations with prior COVID-19 status were identified for sex (*z* = −9.58; *P* < .001), income (*z* = 9.75; *P* < .001) Black vs White race (*z* = 3.02; *P* = .003), and urban vs rural locale (*z* = 2.89; *P* = .004) (Figure 1). Comparing individual symptoms among those with or without prior COVID-19, the greatest differences in PHQ-9 scores were observed for suicidality (mean [SD], 2.50 [1.06] vs 1.99 [1.09]; *t* = −24.83; *P* < .001) and motor symptoms (mean [SD], 2.58 [1.01] vs 2.11 [1.06]; *t* = −23.74; *P* < .001) (Figure 2). The risk for depressive symptoms increased with greater duration after acute illness (for each additional month, crude OR, 1.07; 95% CI, 1.05-1.09; adjusted for survey month, state, and sociodemographic features, adjusted OR, 1.09, 95% CI, 1.07-1.11).

## Discussion

In this survey study, the magnitude of depressive symptoms and sociodemographic variables differed between individuals with and without prior COVID-19 illness. These differences in phenomenology and risk factors both indirectly suggest that apparent major depressive episodes following COVID-19 illness may be distinct from those typically observed in adults. Furthermore, the risk for depressive symptoms increased with greater postacute interval, rather than the gradual reduction anticipated if depressive symptoms are a consequence of acute increase in illness-associated stressors. In interpreting our results, we note the key limitations that the PHQ-9 samples only a subset of symptoms and that details of psychiatric history and COVID-19 course are not available, necessitating confirmation in longitudinal cohorts.

The difference in symptom profile is notable in light of the markedly elevated rates of delirium observed in patients with COVID-19,^5,6^ given that delirium is also often associated with both motoric and cognitive symptoms. On the other hand, the lesser differences in concentration and fatigue suggest that post–COVID-19 depressive symptoms are not entirely explainable by other postacute systemic symptoms.

Broadly, our results may suggest a different disease process at least in a subset of individuals. At minimum, these distinctions suggest a need to better understand differences and similarities between depressive symptoms following COVID-19 and those associated with typical major depressive disorder.
